# Single-shot spectro-temporal characterization of XUV pulses from a seeded free-electron laser

**DOI:** 10.1038/ncomms9075

**Published:** 2015-08-20

**Authors:** Giovanni De Ninno, David Gauthier, Benoît Mahieu, Primož Rebernik Ribič, Enrico Allaria, Paolo Cinquegrana, Miltcho Bojanov Danailov, Alexander Demidovich, Eugenio Ferrari, Luca Giannessi, Giuseppe Penco, Paolo Sigalotti, Matija Stupar

**Affiliations:** 1 Sincrotrone Trieste, Trieste 34149, Italy; 2 Laboratory of Quantum Optics, University of Nova Gorica, Nova, Gorica 5001, Slovenia; 3 Laboratoire dOptique AppliquÂee, UMR 7639, ENSTA-CNRS-Ecole, France; 4 Università degli Studi di Trieste, Dipartimento di Fisica, Piazzale Europa 1, 34100 Trieste, Italy; 5 Theory GroupENEA C.R. Frascati, Via E. Fermi 45, 00044 Frascati, Italy

## Abstract

Intense ultrashort X-ray pulses produced by modern free-electron lasers (FELs) allow one to probe biological systems, inorganic materials and molecular reaction dynamics with nanoscale spatial and femtoscale temporal resolution. These experiments require the knowledge, and possibly the control, of the spectro-temporal content of individual pulses. FELs relying on seeding have the potential to produce spatially and temporally fully coherent pulses. Here we propose and implement an interferometric method, which allows us to carry out the first complete single-shot spectro-temporal characterization of the pulses, generated by an FEL in the extreme ultraviolet spectral range. Moreover, we provide the first direct evidence of the temporal coherence of a seeded FEL working in the extreme ultraviolet spectral range and show the way to control the light generation process to produce Fourier-limited pulses. Experiments are carried out at the FERMI FEL in Trieste.

## Abstract (web summary)

X-ray free-electron laser is a power probe for materials, but it is challenging to measure the spectro-temporal characters of individual pulses. Here, De Ninno *et al.* implement an interferometric method allowing one to characterize and control the ultrashort XUV pulses seeded by a femtosecond laser.

## Graphical abstract

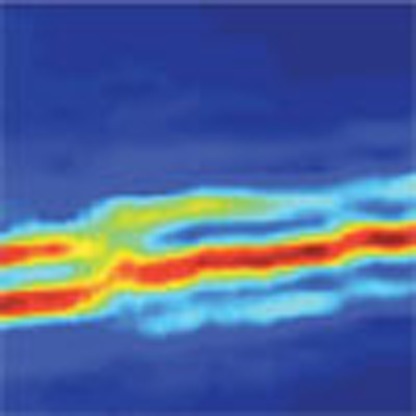

The availability of ultrashort fully coherent pulses generated by seeded free-electron lasers (FELs)^1^,^2^,^3^,^4^,^5^,^6^,^7^,^8^,^9^ in the ultraviolet and extreme ultraviolet (XUV) spectral ranges opens the door to completely new experiments of nonlinear optics^10^,^11^. Moreover, it allows the possibility of extending the concepts of coherent quantum control to short wavelengths^12^,^13^ and of realizing the potential of numerous advanced spectroscopic techniques, such as resonant inelastic X-ray scattering^14^ and coherent ultrafast core-hole correlation spectroscopy^15^. While spatial coherence is also a property of FELs based on self-amplified spontaneous emission^16^, the capability of generating temporally coherent pulses is a distinctive feature of seeded FELs. Indeed, this is a natural consequence of the principle on which a seeded FEL relies (Fig. 1): before emitting radiation, electrons interact with a coherent source (the seed) and, under given conditions, the latter transmits its coherence properties to the FEL light.

In addition to amplification, several factors contribute to the evolution of the electric field during the FEL process. A linear frequency chirp d*ω*/d*t* in the seed affects the emission process, causing a broadening of the spectral envelope^17^. Furthermore, before interacting with the seed, electrons are accelerated close to the speed of light. During acceleration and transport, the electron beam is exposed to sinusoidal radio-frequency electric fields and wakefields and therefore acquires a time-dependent energy profile. A curvature d^2^*E*(*t*)/d*t*^2^ in the electron energy *E*(*t*) produces the same effect as a linear frequency chirp in the seed^18^,^19^,^20^ and causes an additional linear frequency offset during amplification due to varying d*E*(*t*)/d*t* along the electron beam^21^. As shown below, such an offset plays a key role in the method we have implemented for the reconstruction of the FEL pulse. The interplay between these effects determines the FEL temporal phase, which can in turn drastically modify the spectral content of the radiated light^22^.

Single-shot characterization and control of the spectral and temporal features of FEL pulses are fundamental prerequisites for experiments aiming at taking full advantage of the laser-like properties of a seeded FEL. In fact, such a characterization is a challenging task, mainly due to the strong absorption of XUV light in solid-state crystals and to the ultrashort duration of the pulses under scrutiny. Until now, only few effective techniques have been proposed to measure the duration of a femtosecond short-wavelength self-amplified spontaneous emission FEL pulse^23^,^24^.

In this letter, we take a step further and demonstrate the possibility to reconstruct, both in the temporal and spectral domains, the envelopes and phases of a pulse generated by a seeded FEL. The proposed method is based on the generation of two FEL pulses and on their characterization through spectral phase interferometry for direct electric-field reconstruction (SPIDER)^25^,^26^,^27^,^28^,^29^. Our results demonstrate the possibility to take advantage of the interplay between the seed and electron-energy chirps to generate Fourier-limited pulses, that is, pulses with the minimum possible time–bandwidth product.

## Results

### Generation of the interferogram

**Figure 1:**
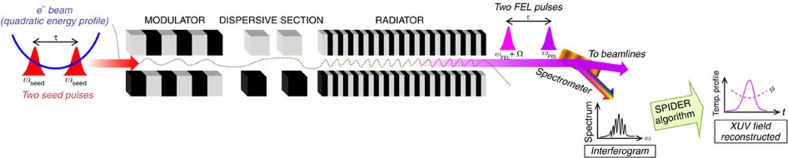
The seeded FEL scheme. Before emitting radiation during their transit through a long undulator (the radiator), electrons interact with a coherent light source (the seed) in a shorter undulator (the modulator). The coherent seed imparts an energy modulation to electrons, which is transformed into a density modulation (bunching) when the electrons, before injection into the radiator, cross the magnetic field generated by a dispersive section. The bunching has a significant harmonic content at the frequency of the seed, *ω*_seed_, and at its low-order harmonics. The radiator is tuned to the *n*th harmonic of the seed and the emission occurs at the frequency *ω*_FEL_=*nω*_seed_. In the reported experiments, the seed consisted of two identical laser pulses at 261 nm, separated by a temporal distance *τ*=230 fs; moreover, *n*=5. The seed replicas interact with a portion of the electron beam characterized by a quadratic energy chirp. At the end of the radiator, this results in the generation of two identical FEL pulses separated in time by *τ* and shifted by a frequency Ω, which is directly proportional to the amount of electron-energy quadratic chirp. The two FEL pulses generate a spectral interference pattern, which is acquired using the FERMI spectrometer^30^ and analysed off-line by means of a SPIDER algorithm (see Methods). The analysis allows to reconstruct the spectro-temporal properties of the FEL pulse.

**Figure 2:**
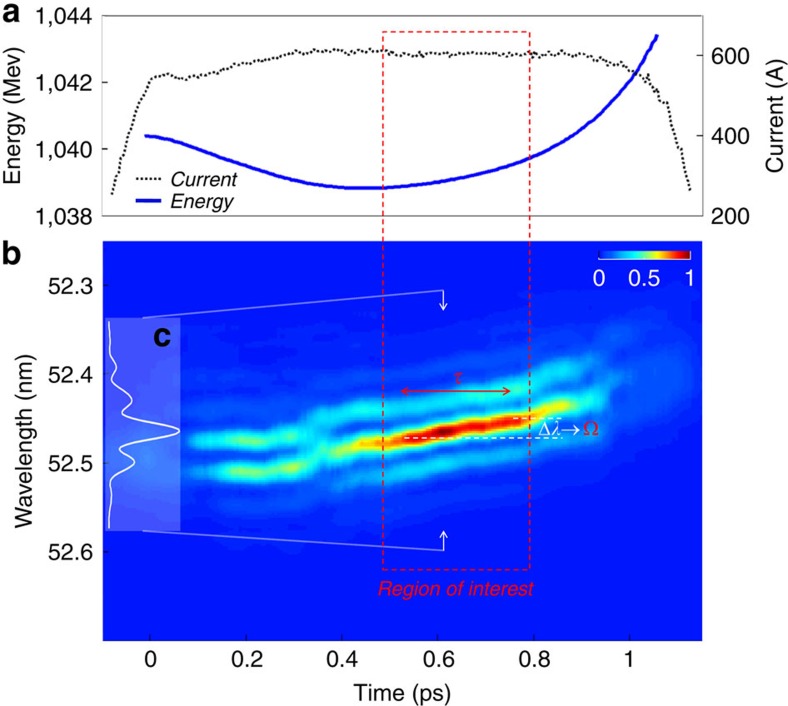
Generation of the FEL interferogram. (**a**) Energy and current time-dependent profiles of the FERMI electron beam (the bunch head comes at smaller times). In the highlighted region, the energy profile is characterized by a dominant and constant quadratic term. (**b**) FEL intereferograms as a function of the temporal position of the leading seed pulse with respect to the electron beam. The region of interest corresponds to the overlap between the seed replicas and the electron-beam quadratic region. Also shown is the definition of the parameters *τ* and Ω. For the reported experiments, Ω=1.7 × 10^13^ Hz. (**c**) Vertical cut of the map, providing a single-shot interferogram.

As mentioned, the electron-beam energy is generally characterized by a time-dependent profile. If two seed replicas separated by a time interval *τ* overlap with a quadratic zone of the *E*(*t*) curve, two FEL pulses are generated (see Fig. 1), which are separated by the same temporal distance and shifted in frequency by a shear Ω depending on the quadratic curvature. Moreover, for a sufficiently homogeneous electron beam (that is, constant emittance, energy spread and current) and a properly tuned FEL, the generated pulses have equal intensities and temporal phases. These are the conditions realized at FERMI and needed for carrying out the SPIDER analysis. A fine-tuning of the accelerator parameters allows the generation of stable and reproducible charge distributions with homogeneous properties, see for example the current profile in Fig. 2a, where a plot of the measured electron-beam energy profile is shown. The highlighted region is characterized by a dominant quadratic dependence of *E*(*t*). In such a region, higher-order nonlinear terms are negligible. Importantly, the amount of quadratic chirp can be controlled by adjusting the parameters of the linear accelerator (see Methods).

The twin FEL pulses yield a spectral interferogram on which the SPIDER algorithm relies. As shown in Fig. 2b, the interferograms evolve as a function of the temporal position of the seed replicas with respect to the electron beam. Vertical cuts of the map provide single-shot interferograms, see Fig. 2c. The good contrast of the interference fringes is a clear indication of the similarity of the interfering pulses. Moreover, it provides a direct demonstration that temporal coherence imprinted onto the electrons by the seed is conserved during the FEL process. As expected, the central wavelength of the inteferograms generated by the electrons in the quadratic region displays a linear shift. These interferograms are located in the region of interest highlighted in Fig. 2b. Note that in this region the fringes in the interferograms do not evolve significantly. This is a nice proof of phase locking between the two pulses and a confirmation of the homogeneity of the electron beam. The measured shift between spectra separated by the temporal distance *τ* corresponds to the spectral shear Ω. For the reported case, the two seed pulses have a duration of 125 fs (full-width at half-maximum (FWHM)) and a positive linear frequency chirp (see Methods).

### Analysis of the interferogram

**Figure 3:**
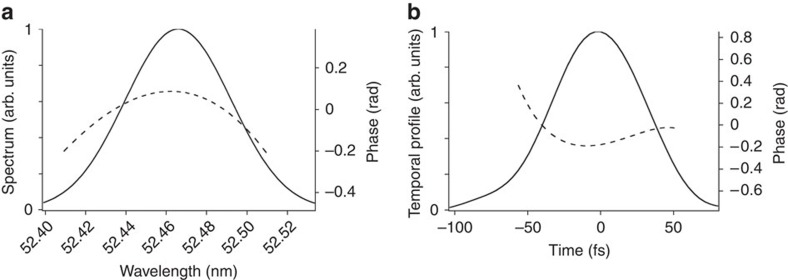
Spectro-temporal reconstruction of an FEL pulse. (**a**) Reconstruction of the spectral envelope and phase from an interferogram acquired in the middle of the region of interest shown in Fig. 2b. (**b**) Reconstruction of the temporal envelope and phase. Due to the constraints in the choice of the working conditions (see Methods), the tails of the temporal phase may be affected by spurious high-frequency terms. For this reason, the phase plots are truncated before the possible appearance of the artificial distortion (see also Fig. 4).

The inteferograms in the region of interest were used to carry out the SPIDER analysis of the FEL pulse. Figure 3 shows the result of the reconstruction in the spectral (Fig. 3a) and temporal (Fig. 3b) domains for a particular interferogram located in the middle of the region of interest. The temporal phase profile has a positive curvature, corresponding to a dominant positive linear frequency chirp on the pulse. This is consistent with the fact that both the electron-beam energy and the seed pulses are characterized by a positive chirp. The obtained spectral bandwidth and pulse duration are, respectively, 6.3 × 10^−2^ nm and 71 fs (FWHM). The value of the bandwidth is very close (within a few per cent) to the one obtained by directly measuring the spectrum of a single FEL pulse, generated by one of the seed replicas. According to the existing theory^20^, the FEL pulse duration, Δ*t*_FEL_, is expected to scale as Δ*t*_seed_/*n*^1/3^ (where Δ*t*_seed_ is the duration of the seed pulse), that is, 73.7 fs (FWHM) for the conditions of the reported experiment. Our measurement shows a remarkably good agreement with the theoretical prediction. The time–bandwidth product of the pulse is a factor of 1.1 above the Fourier limit.

### Reproducibility of the spectro-temporal reconstruction

**Figure 4:**
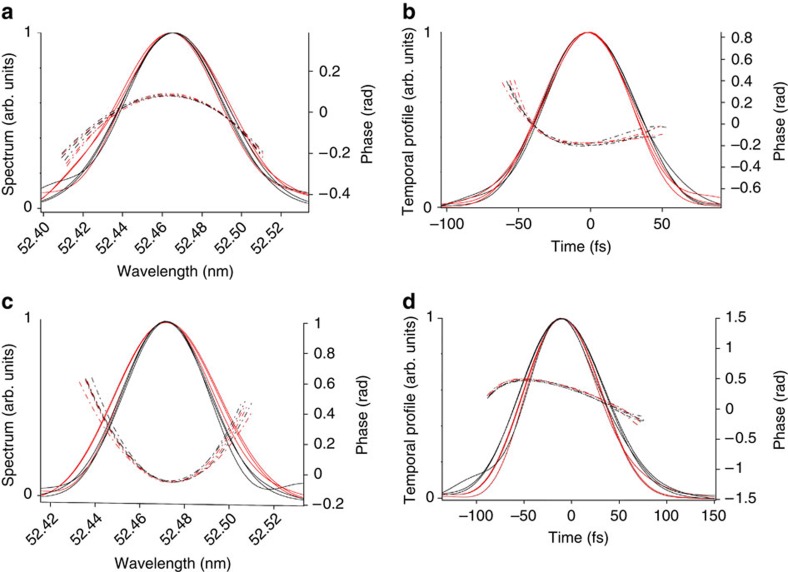
Statistical analysis of spectro-temporal reconstructions. (**a**,**b**) Positive linear frequency chirp on the seed replicas (quadratic temporal phase: about 1 × 10^−5^ fs^−2^): Reconstructions obtained from three consecutive FEL shots (lines of the same colour) at fixed delay and for two different delays (represented by black and red colours) separated by 65 fs within the region of interest shown in Fig. 2b. Continuous line: spectral and temporal envelopes; dotted lines: spectral and temporal phases. (**c**,**d**) Negative linear frequency chirp on the seed replicas (quadratic temporal phase: about −4.5 × 10^−5^ fs^−2^): Reconstructions obtained from three consecutive FEL shots (lines of the same colour) at fixed delay and for two different delays (represented by black and red colours) within the region of interest shown in Fig. 2b. Continuous line: spectral and temporal envelopes; dotted lines: spectral and temporal phases. For the sake of visualization, spectra were centred at the same wavelength. Due to the reasons explained in the caption of Fig. 3 (see also Methods), the tails of the phase plots are truncated before the possible appearance of the artificial nonlinear terms.

The reproducibility of our method is demonstrated by comparing the reconstructions obtained from consecutive FEL shots and for different positions inside the region of interest. The results are shown in Fig. 4a,b: the lines of the same colour represent the reconstructions obtained, at a given delay, from three consecutive interferograms, while different colours correspond to different delays. The reconstructions are very similar and fluctuations of the bandwidth and pulse duration are smaller than 10%, confirming the excellent stability of the FERMI FEL.

The experiment was repeated with two seed replicas characterized by a negative linear frequency chirp and a duration of 180 fs (FWHM). The results are shown in Fig. 4c,d. The spectral and temporal phases change concavity with respect to the previous case. This is a consequence of the fact that now the electron-beam energy and the seed frequency chirps have opposite signs, with the effect of the chirp on the seed being predominant. The spectral bandwidth, 4.8 × 10^−2^ nm, is again close to that of a single FEL pulse, while the FEL pulse duration, 99 fs, corresponds well to the theoretical prediction of 105.3 fs. Also in this case, the fluctuations of the bandwidth and pulse duration are smaller than 10%. The calculation gives a time–bandwidth product that is a factor of 1.2 above the Fourier limit.

### Control of spectro-temporal properties

A comparison between the results reported in Fig. 4 for the two different seed conditions provides a clear evidence of the possibility to fully control the FEL spectral and temporal properties by properly adjusting the seed laser chirp. This opens the way to the generation of flat-phase Fourier-limited femtosecond pulses in the XUV region using a seeded FEL.

## Discussion

The method we have implemented allows to carry out single-shot spectro-temporal characterizations of femtosecond XUV pulses produced by a seeded FEL. Our measurements also constitute the first direct evidence of the temporal coherence of a seeded FEL pulse. Moreover, we provided a clear indication of the strategy for generating Fourier-limited FEL pulses. Our results are supported by, and provide support to, previously published theory. The method is independent of the photon energy and decoupled from machine parameters. Therefore, it can be easily implemented on present and future facilities, providing users with the unique possibility to monitor and shape at will the fundamental properties of a seeded FEL pulse.

## Methods

### Electron-beam, seed and FEL settings

The FERMI electron beam is generated by a photo-injector composed of a radio-frequency (rf) photocathode gun and a booster linac, and it is then accelerated up to 1.0–1.5 GeV by a rf linac. The electron bunch is longitudinally compressed up to 500–600 A by a magnetic chicane. A fourth harmonic rf cavity is routinely used in order to guarantee a linear compression process and therefore a flat current profile at the end of the linac. During acceleration, the electron beam experiences nonlinear wakefields depending on the bunch charge and current profile, and affecting the electron energy profile. By properly adjusting the rf section phases and amplitudes and the compression settings in the chicane, a constant quadratic energy chirp is obtained in the electron-bunch middle core. For the reported experiments, the beam energy was ≃1 GeV, the energy spread ≃150 keV, the slice emittance ≃1 mm mrad and the peak current ≃600 A. In these conditions, the core of the electron beam is characterized by a dominant quadratic energy curvature of about 6.5 MeV ps^−2^ (see the highlighted region in Fig. 2a).

The seed replicas to generate the twin FEL pulses were obtained by splitting the pulse of the third harmonic of a Ti:Sapphire laser by means of adjustable polarization rotation and a birefringent plate. The plate's thickness defined the distance between the replicas. For these experiments, the seed replicas carried either a positive or a negative linear frequency chirp. The positive chirp was generated by propagating the third-harmonic pulse through a lamina of calcium fluoride; it was verified that, at the used power density level, self-phase modulation effects in the material were negligible. The negative chirp was generated by means of an optical compressor based on transmission gratings. Before injection in the modulator, the spectrum and the duration of the replicas were measured, allowing to infer the amount of linear frequency chirp present in each pulse. The peak power of each seed replica at the entrance of the modulator was about 250 MW, for both chirp configurations.

The FERMI FEL modulator has a period of 100 mm and a length of 3 m. For the reported experiments, the radiator was composed of three 2.4-m-long APPLE-II undulator sections with a period of 55 mm. The strength of the dispersive section was about 50 μm.

### The SPIDER algorithm

Writing the electric field of the non-detuned FEL pulse as *E*(*ω*)=|*E*(*ω*)|exp(*iφ*(*ω*)), the SPIDER signal reads *S*(*ω*)=|*E*(*ω*)|^2^+|*E*(*ω*+Ω)|^2^+2|*E*(*ω*)||*E*(*ω*+Ω)|cos(*φ*(*ω*)−*φ*(*ω*+Ω)+*ωτ*), where *τ* is the temporal distance between the interfering pulses and Ω is the spectral shear. The analysis of the interference pattern allows retrival of the phase *φ*(*ω*) through an inversion routine^26^. The quantity |*E*(*ω*)| can be directly obtained from *S*(*ω*). The temporal profile |*E*(*t*)| and temporal phase *φ*(*t*) can be found by carrying out a Fourier transform of *E*(*ω*).

For the reported experiments, the maximum value of *τ* is determined by the extension of the highlighted region in Fig. 2, where the electron-beam energy profile is characterized by a dominant homogeneous quadratic dependence. The shear Ω is then determined by the amount of such a quadratic chirp. Outside this region, higher-order nonlinear terms become non-negligible. This makes it impossible to generate (almost) identical FEL pulses and, therefore, apply the SPIDER reconstruction. In our working conditions, Ω=1.7 × 10^13^, which corresponds to Δ*λ*=2*π*Ω/*λ*^2^≃0.025 nm (*λ*=52.2 nm). As a consequence, the analysed pulses have been reconstructed, in the spectral domain, using a relatively small sampling. However, as it is mentioned in the body of the article, the obtained result for the spectral bandwidth is in good agreement with the direct measurement carried out with the FERMI spectrometer, which allows one to reconstruct the spectrum with a significantly higher resolution. Moreover, the reconstructed spectral envelope and phase generates (via Fourier transform) a temporal pulse, whose duration is in quite good agreement with theoretical predictions. Based on that, we can safely conclude in favour of the reliability of the reported spectro-temporal reconstruction.

### Diagnostics

The time-dependent electron-beam energy distribution was measured at the end of the linac (see Fig. 2a) by using a radio-frequency deflecting cavity in combination with an energy spectrometer: the beam was vertically stretched by the deflector, horizontally energy dispersed by a dipole and finally intercepted by a YAG screen.

The spectral properties of the FEL were measured using a dedicated spectrometer^30^. The spectrometer uses first-order diffraction while the zero-order beam goes to the experimental station. This allows measurement of the spectrum online, without interfering with the experiments done by the users. The beam is diffracted by a planar variable-spacing grating, which focuses the first-order diffraction onto a YAG screen. The fluorescence intensity is detected by a charge-coupled device (CCD). With this set-up, single-shot spectra can be acquired for FEL intensities higher than a few microjoules.

## Additional information

**How to cite this article:** De Ninno, G. *et al.* Single-shot spectro-temporal characterization of XUV pulses from a seeded free-electron laser. *Nat. Commun.* 6:8075 doi: 10.1038/ncomms9075 (2015).
